# Exploring social harms during distribution of HIV self‐testing kits using mixed‐methods approaches in Malawi

**DOI:** 10.1002/jia2.25251

**Published:** 2019-03-25

**Authors:** Moses K Kumwenda, Cheryl C Johnson, Augustine T Choko, Wezzie Lora, Wakumanya Sibande, Doreen Sakala, Pitchaya Indravudh, Richard Chilongosi, Rachael C Baggaley, Rose Nyirenda, Miriam Taegtmeyer, Karin Hatzold, Nicola Desmond, Elizabeth L Corbett

**Affiliations:** ^1^ Malawi Liverpool Wellcome Trust Clinical Research Programme Blantyre Malawi; ^2^ Helse Nord TB Initiative College of Medicine University of Malawi Blantyre Malawi; ^3^ HIV and Global Hepatitis Department World Health Organization Geneva Switzerland; ^4^ Clinical Research Department London School of Hygiene and Tropical Medicine London UK; ^5^ Department of International Public Health Liverpool School of Tropical Medicine Liverpool UK; ^6^ Population Services International Lilongwe Malawi; ^7^ Ministry of Health Lilongwe Malawi; ^8^ Population Services International Johannesburg South Africa

**Keywords:** HIV/AIDS, HIV self‐test, HIV testing, social harms, Malawi

## Abstract

**Introduction:**

HIV self‐testing (HIVST) provides couples and individuals with a discreet, convenient and empowering testing option. As with all HIV testing, potential harms must be anticipated and mitigated to optimize individual and public health benefits. Here, we describe social harms (SHs) reported during HIVST implementation in Malawi, and propose a framework for grading and responding to harms, according to their severity.

**Methods:**

We report findings from six HIVST implementation studies in Malawi (2011 to 2017) that included substudies investigating SH reports. Qualitative methods included focus group discussions, in‐depth interviews and critical incident interviews. Earlier studies used intensive quantitative methods (post‐test questionnaires for intimate partner violence, household surveys, investigation of all deaths in HIVST communities). Later studies used post‐marketing reporting with/without community engagement. Pharmacovigilance methodology (whereby potentially life‐threatening/changing events are defined as “serious”) was used to grade SH severity, assuming more complete passive reporting for serious events.

**Results:**

During distribution of 175,683 HIVST kits, predominantly under passive SH reporting, 25 serious SHs were reported from 19 (0.011%) self‐testers, including 15 partners in eight couples with newly identified HIV discordancy, and one perinatally infected adolescent. There were no deaths or suicides. Marriage break‐up was the most commonly reported serious SH (sixteen individuals; eight couples), particularly among serodiscordant couples. Among new concordant HIV‐positive couples, blame and frustration was common but rarely (one episode) led to serious SHs. Among concordant HIV‐negative couples, increased trust and stronger relationships were reported. Coercion to test or disclose was generally considered “well‐intentioned” within established couples. Women felt empowered and were assertive when offering HIVST test kits to their partners. Some women who persuaded their partner to test, however, did report SHs, including verbal or physical abuse and economic hardship.

**Conclusions:**

After more than six years of large‐scale HIVST implementation and in‐depth investigation of SHs in Malawi, we identified approximately one serious reported SH per 10,000 HIVST kits distributed, predominantly break‐up of married serodiscordant couples. Both “active” and “passive” reporting systems identified serious SH events, although with more complete capture by “active” systems. As HIVST is scaled‐up, efforts to support and further optimize community‐led SH monitoring should be prioritized alongside HIVST distribution.

## 1 Introduction

Despite concerted efforts to scale‐up HIV testing services, in 2017, approximately 25% of people with HIV remain undiagnosed 1. Globally, men, young people and key populations are disproportionately contributing to this HIV “testing gap” 1. In Malawi, men with HIV are 10% less likely to know their status than women, and only one third of adolescent (aged 15 to 19) boys and less than half of adolescent girls had ever tested 2. HIV testing, prevention and treatment coverage for female sex workers (FSW) also remains suboptimal 3.

HIV self‐testing (HIVST) can increase HIV testing coverage and frequency 4. Several studies in Malawi have shown HIVST to be highly acceptable and able to reach first‐time testers, young people (aged 16 to 25), men and couples and partners 5, 6, with acceptable linkage into facility‐based services when combined with facilitated linkage strategies 6, 7, 8, 9. As with any form of HIV testing, however, potential social harms (SHs) must be anticipated and mitigated 10, 11, 12.

SHs can be defined as any intended or unintended cause of physical, economic, emotional or psychosocial injury or hurt from one person to another, a person to themselves, or an institution to a person, occurring before, during or after testing for HIV 13. SHs are well documented with all HIV testing approaches 14, 15, but need to be balanced against the clear benefits of early treatment and the UNAIDS “90‐90‐90” targets – the first of which is to diagnose 90% of people with HIV by 2020 16.

Couples and partner testing, including HIVST, is a highly effective way to reach those in need of testing, prevention and treatment services 10, 11. Despite the many benefits, coping with serodiscordant results (one partner HIV positive and one partner HIV negative) can be difficult 17, 18. Concerns raised by HIVST include potential misuse, and whether testing without in‐person counselling may exacerbate negative behaviours and adverse consequences 19, 20. An estimated 37% of ever‐partnered women in Africa report having experienced physical and/or sexual intimate partner violence (IPV) 21, and people with HIV, particularly women and adolescents, may have increased risk. Likewise, key populations continue to experience various forms of SHs and violence, including discrimination and criminalization 22.

Despite the concerns, reporting of serious SHs following HIVST appears to be rare 4. Large‐scale evaluations distributing more than one million HIVST kits in three African countries have not identified any suicides 4, 23. Psychological distress following HIVST for those who test positive also appears to be no more extreme than with other approaches to HIV testing, and often short‐term in nature 4, 6, 11, 24. Furthermore, initial SHs can evolve into significant positive outcomes if reviewed in the longer term. Communities and self‐testers also consistently report that access to HIVST is empowering, and that its private nature, in most instances, outweighs possible negative aspects 20, 25.

Beyond clinical trials, efforts to identify and measure SH relating to HIV testing, including HIVST, are limited and not part of routine monitoring. Instead, efforts have focused on mitigation strategies to minimize harms 10, 22. Here, we describe SH events reported during HIVST implementation in Malawi over a six‐year period, propose a community‐led approach for SH monitoring and suggest a framework for grading SHs.

## 2 Methods

Six HIVST implementation studies carried out in Malawi between 2011 and 2017 distributed 175,683 HIVST kits and included 13 different SH substudies (Table 2). Five studies included both qualitative and quantitative components (mixed methods) from the design stage. The sixth study, Partnerships in Self‐Testing in Malawi (PRISM), used a qualitative cohort design nested within a controlled cluster‐randomized trial of HIVST kit distribution (HitTB). Health impacts, including testing coverage, linkage to HIV treatment and prevention, are reported elsewhere. Qualitative methods included focus group discussions (FGDs), in‐depth interviews and critical incident narratives. Quantitative methods included post‐test questionnaires, household surveys, active follow‐up of all deaths and reports of IPV during HIVST implementation. For this analysis, we triangulate from different approaches used over the six studies 26, 27.

The HitTB study (Table 2) was a cluster‐randomized trial implemented in urban Blantyre, distributing 27,789 HIVST kits through trained distributors to 16,660 adult residents (≥16 years) over two years 6, with brief feedback requested from all HIVST participants using a self‐administered questionnaire 6. Outcomes captured at the cluster level included antiretroviral therapy (ART) initiations and deaths 28, with mortality captured through a community reporting system included from the start to capture and report community concerns on a weekly basis. One hundred and twelve “cluster representatives” were recruited with endorsement from community leaders. Cluster representatives reported SH events to a Community Liaison Officer. All deaths among cluster residents (irrespective of HIVST use) were captured through this system, and followed up with verbal autopsies 6. PRISM and Self‐test Impacts (ST‐Impacts) were qualitative substudies recruiting cohorts of self‐testers from HitTB to evaluate broader consequences of HIVST.

PRISM (Table 2) was a qualitative substudy of HitTB that recruited and followed up 67 individuals from 2012 to 2014 25. All participants were cohabitating and in established sexual relationships where either one or both partners had self‐tested. Gender, HIV status, nature of self‐testing (individual vs. couple testing) and test results (concordant HIV positive where both partners are HIV‐positive, HIV‐negative and discordant couples) were used for purposive selection. Self‐tested individuals were interviewed using serial in‐depth interview approach at baseline (within a week of HIVST) and followed up twice within 17 months post‐interview. Five FGDs were also conducted with forty‐three purposively selected community members (twenty women): two exclusively male, two exclusively female and one with male and female participants.

ST‐Impacts (Table 2) recruited 300 HIVST participants from HitTB Study between 2012 and 2013 29. This mixed‐methods substudy compared prospective reports of SHs identified through the community reporting system with those collected through serial biographical interviews, face‐to‐face questionnaires, FGDs, three‐month‐long longitudinal diaries and critical incident narratives.

Partner Assisted HIVST and Linkage (PASTAL) was a separate HIVST trial carried out from 2016 to 2017, recruiting 2349 pregnant women from three urban primary clinics for secondary distribution to male partners (two kits per woman) 9, 30. The primary outcome was linkage to HIV care and prevention services by the male partner. Secondary outcomes were reported by the woman at 28 days, and included safety: women were asked directly about IPV events resulting from delivery and use of HIVST kits using audio computer‐assisted self interviews (ACASI) with all women 28 days after HIVST distribution. Incidents reported by participants through ACASI were followed up, documented onto standardized forms and classified by a qualitative researcher probing the nature and relatedness to HIVST of the incident.

For PASTAL, a framework was developed for adverse events reporting, focused on IPV and self‐harm 9, 13 and further adapted to Table 1. The approach used standard pharmacovigilance reporting 31 that defines potentially life‐threatening/changing events as “serious,” and events with no or some effect on social‐ and work life as “mild” or “moderate” respectively. HIVST studies reporting data from earlier time periods did not systematically capture the data relating to life impact needed to classify severity, and so may have misclassified some serious events.

**Table 1:** Proposed social harms grading matrix: adapted from Division of AIDS, and revised following use in three studies, including Self‐Test Africa Research general population and female sex workers protocols

Grade 1 (mild) No effect on social and work life. No doctor needed	Grade 2 (moderate) Some effect on social or work life, and may need doctor or psychologist	Grade 3 (severe) Unable to socialize or unable to work, and needs doctor or psychologist	Grade 4 (life‐threatening) Life‐threatening/disability Grade 5: fatal
Denying access to non‐critical household resources Being ignored Being controlled (e.g. not allowed to leave house) Being shouted at	Moderate verbal, emotional or psychological IPV Coercion to self‐test Coercion to disclose a self‐test result IPV that includes, e.g. pushing or slapping with an open hand that does not result in pain or visible marks >24 hours Psychologically coercive sex Being shunned at home, work or school Economic hardship resulting in skipping meals, missing school Temporary separation lasting less than seven days	IPV that leads to pain, bruising or marks >24 hours. Verbal threats of potentially lethal violence (e.g. statement of intent to kill, mock strangulation, threatened with a knife or gun) Marriage break‐up lasting greater than or equal to seven days (temporary or permanent) Stigmatization sufficient to cause change of work, school or home Suicidal ideation Extreme economic stress: unable to meet basic needs of self/children	IPV leading to hospitalization Attempted suicide leading to hospitalization Attack using potentially lethal force (e.g. knife, gun, hammer, kicks to head, asphyxiation) Rape or attempted rape *[Any event leading to death is classified as a Grade 5 serious SH]*
Referred to community‐based institutions for assistance, for example CBOs, Police.	Refer to community‐based institutions for assistance Reported to relevant authorities, for example Community Liaison Officer Refer to community‐based GBV support organizations	Report to marriage counsellors Report to relevant authorities, for example Community Liaison Officer Refer to community‐based GBV support organizations	Discuss and refer to police/chief/other social support based on individual need and desire Report mandatory events to police (suicide/homicide) Report to relevant authorities, for example programme managers Refer to community‐based GBV support organizations Ensure safe alternative abode before discharge

IPV, intimate partner violence; CBOs: community‐based organizations; GBV: gender‐based violence.

**Figure 1:**
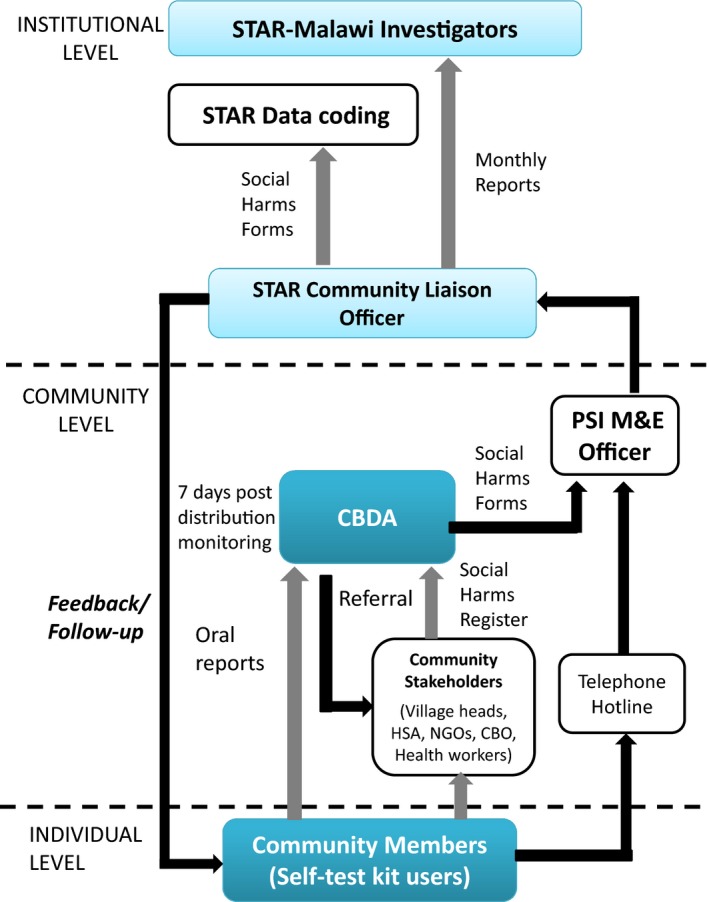
Self‐Test Africa Research (STAR) general population community‐led social harm tracking system, based on engagement of existing authorities and civil society organizations to provide a community‐led reporting system PSI, Population Services International (implementing organization in STAR‐Malawi); M&E, Monitoring and Evaluation; CBDA, community‐based distribution agent; HSA, Community Health Worker cadre of Ministry of Health, Malawi; NGO, non‐governmental organization; CBO, community‐based organization.

As part of the Self‐Testing Africa Initiative (STAR), a community‐based cluster‐randomized trial in general populations (GP) (STAR‐GP) 32 and a mixed‐methods study among FSWs (STAR‐FSW) 33, as described in Table 2. Across both, SHs were actively monitored (Figure 1) and graded using the adapted PASTAL framework that included stigma‐related events (Table 1).

**Table 2:** Studies with nested qualitative data collection on SHs

Study	HIVST strategy	Methodology	Study year/publication	Population: nature	HIVST clients	Adverse event N and %		Serious SHs: N and %		Comment
A: Active SHs identification systems (research)										
1. HitTB	Community‐based	Representatives reporting deaths & community views on HIVST	2011 to 2014 Choko 2015 6	All adults in HIVST area Years 1 and 2	27,789^a^	NA	NA	0	0.0%	No deaths related to HIVST from 132 deaths with verbal autopsy. No serious events identified through Community liaison system, but not focused on IPV
2. HitTB (subset of kits listed above)	Community‐based	Self‐completed post‐HIVST questionnaire for coercions	2012 to 2014 Choko 2015 6	Self‐testers Year 1 data	10,017^b^	288	2.9%	NA	NA	Questionnaire asked only if “Forced to test”: of coerced self‐testers, 94.4% were still satisfied with and would recommend HIVST to others
3. PRISM (substudy of HitTB)	Community‐based	Cohort with serial interview	2012 to 2013 Kumwenda 2014/2018 25, 49	Self‐testers	67^b^	NA	NA	5	7.5%	Purposive selection: all in stable relationship; over‐representation of discordant couples
4. ST‐Impacts (substudy of HitTB)	Community‐based	Cohort with serial interview	2013 to 2015	Self‐testers	300^b^	NA	NA	4	1.3%	Purposive selection: 100 people in established couples, 100 single men and 100 single women
5. ST‐Impacts (substudy of HitTB)	Community‐based	Critical incident narratives: all reported IPV)	2013 to 2015	Self‐testers	13,785^b^	15	0.1%	2	0.01%	All women reporting IPV to HIVST distributor, HitTB Community Representatives, police, support groups, marriage counsellors: from 150 interviewed, 15 reported links to HIVST
6. PASTAL	Antenatal: Partner‐delivered	Interviews using ACASI, 28 days post intervention	2016 to 2017 Choko 2019 9	Pregnant women	4698^a^^,^ d	3[3]^d^	0.1%	0	0.0%	Woman given two HIVST kits. Systematic ACASI capture reflects primarily woman's experience
7. STAR‐GP	Community‐based	Survey of rural villagers	2016 to 2017 Indravudh 2019 54	Self‐testers	794^b^	4	0.5%	NA	NA	Endline survey in randomly selected households of HIVST Evaluation Villages
8. STAR‐FSW: Blantyre	Network‐based	Cohort peer‐reporting system	2017 to 2018	Self‐testers	2001^a^	1	0.0%	1	0.05%	Serial interviews and Longitudinal Diaries conducted but not yet analysed: provisional data
B: Community reporting systems										Facilitated and formalized passive reporting
9. STAR‐GP: HIVST evaluation villages	Community‐based	Community‐led Reporting System established for HIVST	2016 to 2017 Indravudh 2018 54	Self‐testers	9492^a^	ND	ND	6	0.06%	Existing authorities and civil society groups engaged to establish a community‐led system for harms reporting and management (Figure 1)
10. STAR‐GP: Control villages	No HIVST	Community‐led reporting system for standard HTS		Facility HIV testers	3150^c^	NA	NA	[6]^e^	0.19%	Related to standard HIV testing, not HIVST. Same system as used for STAR HIVST evaluation villages
C: Other SHs identification systems										
11. STAR‐GP: non‐evaluation villages	Community‐based	No community‐led reporting system established	2016 to 2017	Self‐testers	128,423^a^	NA	NA	1	0.00078%	Harms reporting relied on HIVST implementers, without establishing community‐led system
12. STAR‐FSW: routine	Network‐based	Routine reporting to implementer	2017 to 2018	Self‐testers	3280^a^	ND	ND	0	0.00%	Not included in Serial interviews and ACASI
13. STAR‐FSW peer distributors	Not Applicable	FGDs	2017 to 2018	Distributors	17^c^	3	17.6%	0	0.00%	Not anticipated or captured systematically: reported at FGDs to evaluate distributor experience
Total HIVST and serious SHs reported in Malawi			2011 to 2018	Self‐testers	175,683^a^			19	0.011%	Affected individuals (total of 25 serious SHs)

^a^A primary study contributing to the total number of 175,683 self‐test kits distributed; ^b^A subset/substudy of an already included primary study (not contributing to the total number of 175,683 self‐test kits distributed); ^c^Study component for which no HIVST kits were used (control or distributor data); ^d^4698 HIVST kits provided to 2349 pregnant women, intended for use by the woman plus her main male partner. 3[3]: women were directly interviewed for SHs using ACASI of whom three reported an adverse event that also affected three male partners – none of whom reported the event spontaneously; ^e^[6] SHs related to standard HIV testing for residents of control villages where self‐test kits were not distributed. Studies: HitTB: Cluster‐randomized trial of health outcomes from introducing a community‐based HIVST distribution strategy; PRISM: Partnerships in Self‐Testing in Malawi; qualitative substudy of HitTB HIVST participants; ST Impact: Self‐test Impact; qualitative substudy of HitTB HIVST participants; PASTAL: Partner Assisted HIVST and Linkage; cluster‐randomized trial of six different approaches to providing HIV testing and encouraging linkage to post‐test services for male partners of antenatal clinic attendees; STAR‐GP: Self‐Test Africa Research – general population protocol; STAR‐FSW: STAR – female sex workers protocol. ACASI, audio computer‐assisted self interviews; FGDs, focus group discussions; HIVST, HIV self‐testing; HTS, HIV testing services; IPV, intimate partner violence; NA, not applicable; ND, not determined; SH, social harms.

In STAR‐GP 32, community‐led SHs reporting was introduced into 22 villages (11 HIVST and 11 standard testing services). Pre‐existing community structures (village heads, police, community health workers, religious leaders and marriage counsellors) were responsible for identifying and reporting harms relating to HIV testing. Community leaders documented, investigated, managed and reported SH episodes to the study's Community Liaison Officer. In HIVST clusters, distributors promoted HIVST kits and other health‐related products. Reported SHs from distribution of 137,915 HIVST test kits in four rural districts are listed according to the nature of the reporting system under which they were captured in Table 2 (see Rows 7, 9 and 11). Qualitative process evaluation data were collected during and after HIVST distribution, including six FGDs with fifty healthcare workers, two with eighteen SH reporting‐systems members from “evaluation villages” (see Row 9 of Table 2) and forty‐six in‐depth interviews with HIVST distributors and self‐testers. Evaluation villages were selected to be representative of the wider STAR‐GP distribution model, and had the same implementation strategy, but more intensive monitoring, but a more active community‐led SH reporting system (Figure 1) and endline household surveys 32. Determination of the severity of reported SHs was mostly based on researcher's opinion after a critical analysis of the event.

STAR‐FSW (Rows 8, 12 and 13 of Table 2) assessed the distribution of 5281 HIVST kits in three districts (Blantyre = 2001, Chikwawa = 1237 and Mulanje = 2043). All kits were distributed to FSW by trained FSW who served as peer educators. Implementation, including SH, was monitored using a combination of peer‐led community reporting system, ACASI (n = 268), longitudinal diaries, serial biographical interviews (n = 22), and four FGDs among FSWs (n = 3) and peer distributors (n = 1). Active SH monitoring was only implemented in Blantyre district. Only the peer‐led community reporting system data, ACASI and FGDs are presented.

### 2.1 Data analysis

Detailed descriptive scripts on episodes of SH were reviewed and compared across studies. MK‐coded qualitative data according to the nature (category) of the incident (e.g. divorce/separation, physical violence, verbal abuse, etc.) and groups affected (i.e. men, women, couples, sex workers). Manual coding was used because the datasets on SH were small for any given study. Categories were defined purposively and deductively, to provide overarching guidance on both the nature of SHs within Malawi, and the groups of people who are likely to be susceptible.

While the majority of kit recipients across all studies reported positive experiences and benefits of HIVST 8, here we focus on SHs. Findings are presented as summary frequency tables, and using descriptive narrative supported by relevant quotes from those reporting and experiencing harms. As the focus of data collection and/or reporting was on serious SHs, we have not included estimates of the frequency or severity of all mild and moderate SHs here, with the exception of coercive testing and temporary separation of couples where data was systematically captured.

### 2.2 Ethical considerations

All studies were approved by College of Medicine Research and Ethics Committee of the University of Malawi, and either London School of Hygiene and Tropical Medicine or Liverpool School of Tropical Medicine (ST‐Impacts). All participants provided informed consent as per parent study requirements.

## 3 Results

Between 2011 and 2017, a total of 175,683 HIVST kits were distributed and 25 reported SH events were (0.011%) classified as serious SHs (Table 3). During this period, there were no reported deaths, suicides or incidents of self‐harm, although one man had suicidal ideation (Table 3). Of the twenty‐five serious SH events reported, most were marriage break‐ups (sixteen individuals; in eight couples). In all studies, there was the disproportionate risk of separation when HIV serodiscordancy was newly identified by HIVST. Out of eight marriage break‐ups reported, all but one was in a serodiscordant relationship. The other break‐up was among a concordant HIV‐positive couple.

**Table 3:** Summary of serious social harms (SHs), by nature of harms and whether related to HIV serodiscordancy or not

Nature of event	Individuals affected	Couples affected	Of couples: with HIV discordancy	Total serious SHs
Break‐up of marriage/cohabiting couple	16	8	7	20^a^
Resolved (after at least seven days separation)	8	4		
Unresolved	8	4		
IPV with temporary less than seven days separation	1	1	0	1
Suicidal ideation	1	1	1	1
Use of HIVST kit by 12‐year‐old girl with previously undisclosed and untreated perinatal‐acquired HIV infection	1	0	NA	3^b^
Total with at least one serious SH	19	10	8	25

^a^One break‐up with four individual serious SHs (two individuals affected by marriage break‐up; woman subject to violent assault including a broken arm; woman left in extreme economic hardship). Two break‐ups with three individual serious SHs (two individuals affected by each marriage break‐up; both women subject to extreme economic hardship); ^b^Girl tested in front of school friends and experienced severe stigmatization, psychological distress and economic upheaval with family moving to a new village. Additional family members are likely to have experienced serious SHs but these were undocumented.

Separating couples were more likely to report additional SHs related to physical IPV, or economic hardship, compared to individuals or other couples that stayed together. Except for serodiscordant couples, individuals with history of violence in their relationship were more likely to report experiencing SH than other groups. Pre‐existing violence, prior to HIVST, such as verbal insults and physical violence were frequently experienced by FSWs, but with few instances directly related to HIVST. Economic hardship was rarely reported outside of the context of impending separation/marriage break‐up.

Couples also reported HIVST had many benefits, suggesting it helped facilitate important discussions, built trust and enhanced partner fidelity and increased efforts to jointly reduce sexual risk behaviour. In particular, women reported HIVST was empowering, made them feel in control testing environment and provided new opportunities to discuss testing with their partners (Table 4, Q1 and Q2).

**Table 4:** Quotes on episodes of social harms from six studies from 2011 to 2017 in Malawi

Theme	Number	Quote
Social benefits	Q1	“Our relationship has changed because we are having the same mind.” ST‐Impacts: Woman who tested as a couple, concordant negative
	Q2	“Because it's like you are now open to one another, everyone knows each other's status. But also, it helps that you should be open to one another.” ST‐Impacts: Woman who tested individually, negative
Coercion to test and disclose	Q3	“It is sometimes good … if one of you in the relationship is refusing to get tested you can doubt them. It is good at times to force someone to get tested so that you all know your HIV status. For someone like me who isn't married there is no reason to be forced to get tested.” ST‐Impacts: individual man who self‐tested
	Q4	“It is necessary because they are wishing you well. People must know how they are (HIV status) before it is too late. It becomes very sad when people get really sick and yet all along their friends were telling them to get tested.” ST‐Impacts: individual woman who self‐tested negative
	Q5	“When I got the kit, I took two days without testing, then my wife said that I won't eat that day If I don't test. She went to the bedroom and poured water on my clothes. There was force, I knew that if I don't test then there won't be sex for me.” ST‐Impacts: Husband in a concordant negative couple
Verbal abuse	Q6	“That is when he self‐tested negative. From that moment, I did not understand that he did not have the HIV. That day, it was not a nice experience for me. He was shouting at me; ‘you are a liar’. There is something that you have been doing behind my back.” PRISM: Female, 32 years, HIV‐positive discordant
	Q7	“My trust in you has now eroded and when I look at you now … I now see you as a monster because you have damaged my body [infected her with HIV].” PRISM: Female, 24 years, HIV‐positive concordant
	Q8	“They face FSWs that don't want to test. Most FSWs say bad things to PDs for example swearing at them for approaching them with the kit.” STAR‐FSW: Peer distributor, FGD
	Q9	“They were insulting us, saying no FSW is negative. My neighbours were saying I am HIV positive that is why I was distributing the kits.” STAR‐FSW: Peer distributor, FGD
	Q10	“Neighbours were rude to us asking questions like are you a doctor? Did you go to school?” STAR‐KP: Female, Peer distributor, FSW, FGD
Physical violence	Q11	“I couldn't have gone through this (the beating) if it weren't for self‐testing. I know my husband is very angry right now because I put him through self‐testing and he was found positive.” ST‐Impacts: Wife (negative) in discordant relationship
	Q12	“At first we were staying normally without any problem before this problem came into existence. I just saw a person start changing his ways and I questioned why he was doing this … All this was happening after getting tested. I didn't experience this before but when I got tested is when I started experiencing violence. When I just do something wrong what he will do is beat me.” ST‐Impacts: Wife (negative) in discordant relationship
	Q13	“Sometimes we women are attacked if we are not listening to what our husbands are telling us to do then they start attacking us. Violence also happen when a man wants to have sex with us and we are refusing. That's violence also, ‘ – it's not right that you should be beaten’ because if he has loved you are supposed to love him back.” ST‐Impacts: Married woman who tested with her partner and was discordant positive
	Q14	“I once had a girl who tried to get her partner tested and he beat her up and left her house. But luckily they worked it out and he returned to the house after some time.” STAR‐KP: Female, Peer distributor, FSW, FGD
	Q15	“I got a report from a girl who was forced by her boyfriend to reveal her results. The guy did not believe her results and he wanted a kit too for himself.” STAR‐KP: Female, Peer distributor, FSW, FGD
	Q16	“A certain girl poured alcohol (Chibuku) on me after telling her that she was HIV positive. However, after everything she apologized and I helped her get medication and we've been friends since then.” STAR‐KP: Female, Peer distributor, FSW, FGD
Separation and break‐up	Q17	“As of now there is nothing easy. As things are now, there is nothing that we can sit down and talk because we don't discuss things, because we cannot even sit down to eat nsima [Staple dish made from maize flour] together. When he comes he eats his nsima in the bedroom and the children and myself we eat here … But when my husband finds money, he keeps it for himself and when I have found mine I have to buy food in the house and everything in the house.” ST Impacts: Wife (positive) in concordant couple
	Q18	“When I left my home to attend a funeral in my home village, he called me when I was planning to return to my house. He said ‘please do not come back. I have married another woman who is now staying with me’. From that time, I have not gone back to my husband.” PRISM: Wife, aged 30, HIV positive from discordant couple
Reaction to Discordancy	Q19	“When we tested, ‘I did not drink water’ [emotionally unsettled] that day. He said ‘we have tested, you have HIV but I do not have it. Where did you get HIV? This marriage will end now and you will soon go to your village’. I sat there speechless. Now we always quarrel because he always speaks demeaning words to me because of my status.” PRISM: 32‐year‐old HIV‐positive wife in a discordant relationship
Treatment‐as‐prevention (ART)	Q20	“Some people when they know that someone has HIV and have started taking ARVs [Antiretroviral] drugs, they feel that they cannot have sex with that person fearing that they can also get infected.” PRISM: 29‐year‐old wife, HIV positive, concordant couple
	Q21	“This medicine (ARVs) that I have started taking I feel it helps protect me since we do not use condoms because we are taking these drugs. These drugs help to protect our bodies from getting more viruses.” PRISM: 26‐year‐old wife, HIV positive in a concordant couple
Suicide threats	Q22	“Even that day [of self‐testing], he was so disappointed and did not even eat or bathe. He told me that while I was sleeping, he went away and planned to kill himself. But after thinking through it, he thought that it is shameful because people would be pointing their fingers at me that my husband has killed himself because of me.” PRISM: 19‐year‐old HIV‐negative wife in discordant relationship
Economic violence	Q23	Interviewer: Is there time that you stop him that he shouldn't buy this, and he accepts not to buy it? PF: No isn't possible, he can't allow that, the way I know him I can't even talk about that. Interviewer: What are you afraid of? PF: I am afraid that we will exchange words. ST Impacts: Married woman tested as couple, negative discordant

FSWs, female sex workers; ST‐Impact, Self‐test Impact; STAR, Self‐Test Africa Research; FGD, focus group discussion; ART, antiretroviral therapy; PRISM, Partnerships in Self‐Testing in Malawi.

### 3.1 “Coercion” to test and disclose

Men and FSW most commonly reported coercion to self‐test. Some degree of coercion was reported by 288/10,017 (2.9%) self‐testers in HitTB – 3.9% in men versus 2.2% in women 6, and by 29/268 (10.8%) FSWs in STAR‐FSW ACASI. Overtly hostile coercion directly following HIVST was not identified. Although uncertain if related to HIVST, there was one case of coercion where a woman reported that her partner had forced her to repeat HIVST to confirm their results were discordant. No FSWs reported they were forced to self‐test or disclose their results by clients or sex partners. However, FSWs reported frequent coercion by employers, facility owners and peer HIVST distributors. The two most commonly reported types of coercion were viewed by HIVST kit recipients as “well‐intentioned” or “socially reasonable,” and neither considered as harmful nor spontaneously reported as “harms.”

The first type of coercion, involving women in long‐term sexual relationships pressurising their male partner to test, was described as “well‐intentioned” (Table 4, Q3 and Q4). Women, viewed as household “custodians of health” in Malawi, indicated that HIVST empowered them to actively promote testing to their male partner, since the discussion was immediate and located within the home (Table 4, Q5). This approach was largely seen during pregnancy where both men and women felt urgency to test. Thus, when pregnant women offered HIVST to their male partners, uptake was high 9, 34. Although uncommon, some incidents of arguments or brief separation were reported (Table 5) but no incidents of physical violence.

**Table 5:** Listing of SHs, focused on serious SHs from self‐testing studies in Malawi as reported through: A. active surveillance (serial interviews, ACASI, surveys), B. Integrated Community Reporting Systems (passive surveillance) and C. other mechanisms

Study	People with ≥1 SHs: n/N (%)	Type of SH	Description	Outcome	Severity grade and number
A: Data collected through active surveillance methods: serial interviews, ACASI, surveys					
PRISM HIVST in GPs, but with discordant couples deliberately over‐represented. 67 individuals 14 couples in seven discordant relationships	4/67 (6.0%) self‐testers with ≥1 SH 2/7 (29%) of discordant couples with ≥1 SH	Marriage break‐up related to confirmed discordancy; plus, verbal and economic IPV	A 32‐year‐old woman tested together with her husband, a 32‐year‐old husband who was employed in the formal sector. The woman tested HIV positive, and the man tested HIV negative. The couple attended for couples‐testing at a primary care clinic, where discordancy was confirmed. The man started to verbally insult his wife, and later abandoned her and their child. The wife was unable to economically fend for herself after being separated from the male partner. Both partners affected	Unresolved	Grade 3 SH × 2 (marriage breakdown) Grade 3 SH × 1 (economic)
		Marriage break‐up related to confirmed discordancy	A 61‐year‐old man tested together with his 30‐year‐old wife and the result were discordant – the wife tested HIV positive. The HIV results were confirmed at clinic‐based HTC. Soon after self‐testing, the woman went to her home village to attend a funeral of her daughter. The male partner took advantage of her departure to marry another woman and in the process abandoning the other woman. Both partners affected	Unresolved	Grade 3 SH × 2
		Suicidal ideation related to confirmed discordancy	A new couple who had been married for five months, self‐tested at home as a couple. The husband tested HIV positive while the wife tested HIV negative, with these discordant results confirmed on retesting at clinic. Subsequently, the husband told the wife that he had been thinking about committing suicide. The wife gave this information to the research team during one of her serial in‐depth interviews	Resolved	Grade 3 SH × 1
ST‐Impacts Serial interview with 300 purposively selected HIVST participants in Blantyre (50 couples; 100 single men; 100 single women)	4/300 (1.3%) self‐testers with ≥1 SHs 2/4 (50%) separation of known discordant couples	Marriage break‐up related to confirmed discordancy Economic IPV	A woman was previously known HIV positive and on treatment, but her new husband refused to believe her, and she stopped taking ART. When self‐testing was introduced the man self‐tested HIV negative, and then brought two kits home for the couple to test together. When this showed discordancy (subsequently confirmed), the man stopped providing for his wife and had several periods of separation, with the marriage unlikely to survive. Both partners affected	Unresolved	Grade 3 SH × 2 (marriage breakdown) Grade 3 SH × 1 Economic IPV
		Temporary separation related to confirmed concordant HIV positive Economic IPV	A man knew himself to be HIV positive and had not disclosed to his wife. She saw him taking medication, however, and so self‐tested herself. Her HIVST result was HIV positive. Following this, the couple tested together and had confirmed concordant HIV‐positive results. The woman was unable to forgive the man for deceiving her about his status, and the couple had periods of separation. The man stopped providing for the woman's children from an earlier marriage	Resolved	Grade 3 SH × 2 (marriage breakdown) Grade 2 SH Economic IPV
ST‐Impacts: critical incident Interview of women reporting IPV to, for example police, women's support organizations) during Year 2 of HitTB in urban Blantyre	2/13,785 (0.014%) self‐testers with ≥1 SH	Physical IPV (woman) and marriage break‐up separation (both) relating to unconfirmed discordant results	A woman self‐tested negative, prompting her partner to self‐test. The man reacted violently to his positive HIVST results, with a severe assault during which the woman sustained a broken arm and was hospitalized. The male partner walked out from the relationship and has not returned	Resolved (admission) Unresolved (marriage)	Grade 4 SH (life‐threatening IPV requiring hospitalization) Grade 3 SH × 2 (marriage breakdown) Grade 3 SH × 1 (economic)
PASTAL: pregnant women ACASI at 28 days, plus self‐reporting to study team	0/4698 (0%) self‐testers with ≥1 SH Six SHs (0.13%) reported by 2349 women distributing 4698 kits Denominator # of discordant couples unknown	Temporary separation after bringing home HIVST kits Verbal IPV	A pregnant woman took two HIVST kits and information leaflets home to her male partner, who reacted angrily and shouted at her for receiving these items without his authorization and knowledge. The man sent his wife to her village but later went to get her. Both partners affected	Resolved within two days	Grade 2 SH × 2 (marriage breakdown) Grade 2 SH × 1 (verbal)
		Temporary separation after bringing home HIVST kits Verbal IPV	A pregnant woman took two HIVST kits and information leaflets home to her male partner, who reacted angrily, saying that this indicated lack of trust on her part. The woman left home for one night before the argument was resolved. Both partners affected	Resolved within one day	Grade 2 SH × 2 (marriage breakdown) Grade 2 SH × 1 (verbal)
		Coerced to test (man); Temporary separation	A pregnant woman took two HIVST kits and information leaflets home to her male partner, who refused to test. The couple argued, and the woman left home for one night. Both partners affected. Woman withdrew from study before ACASI at 28 days	Resolved	Grade 2 SH × 2 (marriage breakdown) Grade 2 SH × 1 (verbal)
STAR‐GP Endline survey	SH in 4 (0.50%) of 794 self‐testers	No severity grade/other details available	On interview of a random sample of 2581 adults living in clusters with CBDAs providing HIVST, 794 said that their last HIV test had been an HIV self‐test. Of these, four agreed that “something bad happened” to them after self‐testing	Not assessed	Not graded

Study	SHs: n/N (%)	Type of SH	Description	Outcome	Severity grade
B: Data collected through integrated social‐harms community reporting systems (passive reporting)					
STAR‐GP Community reporting system during distribution of 9492 HIVST kits to 11 Evaluation Villages provided with community reporting system (CRS)	6/9492 (0.063%) self‐testers with ≥1 SH Distributor training included module on discordancy	Temporary separation related to confirmed discordancy	A woman self‐tested HIV positive, and took a kit for her husband, who self‐tested negative. After confirmatory testing, the husband told the wife to leave their house. She reported this issue to the chief and religious leaders who were part of the SHs reporting system. The two were called to discuss the matter which was resolved within a month and the woman returned. Both partners affected	Resolved within a month	Grade 3 SH × 2
		Temporary separation related to confirmed discordancy	A woman who knew that she was HIV positive and was taking ART decided to use HIVST to disclose her status to her husband. She self‐tested, showed her husband the used kit, and offered him a self‐test kit to use himself. He initially refused, but then self‐tested HIV negative. After confirmatory testing, the husband left the marriage, but the Community Health Worker talked to the husband and encouraged him to support his wife to resume ART. The couple came back together after one month. Both partners affected	Resolved after a month	Grade 3 SH × 2
		Temporary separation related to confirmed discordancy	A woman took two self‐test kits for herself and her husband to test together. At first, the man was reluctant to test, but eventually agreed. He tested HIV positive and his wife was HIV negative. The woman threatened to end the marriage but agreed to seek help from their traditional marriage counsellor (*Nkhoswe*). The discordant HIV results have been confirmed. Both partners affected	Resolved after two months	Grade 3 SH × 2
STAR‐KP: FSWs SHs reporting system included in peer distributor training	1/2001 (0.050%) self‐testers with ≥1 SH	Coerced to disclose	A FSW was forced to disclose her self‐test result by her boyfriend. However, there were no long‐term social or work‐related consequences	Resolved	Grade 2 SH
		Coerced to test (man), physical IPV (woman) and temporary separation (both)	A FSW had self‐tested and wanted her regular partner to self‐test. The partner felt pressurized and reacted violently, hitting the woman (details of severity unclear). The male partner walked out but returned to resume the relationship the following day. Both partners affected	Resolved	Grade 2 SH Grade 3/2 SH (severity unclear) Grade 2 SH × 2
C: SHs reported through other mechanisms (no integrated social‐harms community reporting systems)					
STAR‐GP Reported to PSI during remainder of 137,915 HIVST kits delivered outside CRS	1/120,000 (0.001%) self‐testers with ≥1 SH episode from 120,000 HIVST kits distributed	Psychological distress; withdrawal from school; family relocation Related to underage HIVST and non‐disclose of perinatal HIV	A 12‐year‐old girl obtained a self‐test kit, despite being under‐age (minimum age of distribution under the STAR protocol is 16 years) and self‐tested with friends. In this way, she found herself to be HIV positive. Subsequent clinic counsellor interview showed that she had previously tested HIV positive (presumed mother‐to‐child transmission), but had never been made aware of this, and had never been treated. The girl was shunned by her friends, left school, and the family relocated, leaving their property in their original village. She is now in a new school and has been started on ART with provision of additional post‐test counselling provided by implementers and Ministry of Health personnel. Whole family affected	Partially resolved within a month (back in school) Benefit: ART started	3 SHs × 3 to girl: Psychological distress Stigmatization Economic upheaval Other family members: likely multiple SHs, undocumented
STAR‐KP: FSWs distributors SHs reported during FGDs	No SHs. 3 SHs reported by 3 of 17 (17.6%) distributors	Physical assault of peer distributor	A FSW poured local beer (*Chibuku*) onto a peer distributor in reaction to a positive HIV self‐test result	Resolved	Grade 2 SH
		Verbal assault of peer distributor	A peer distributor was verbally assaulted by her neighbours for distributing self‐test kits. The peer distributor felt sufficiently distressed to be unable to continue working	Unknown	Grade 2 SH
		Verbal assault of peer distributor	A peer distributor was verbally insulted by her neighbours for distributing self‐test kits, who said that she must be HIV positive to be doing this work. The peer distributor felt stressed and demoralized but continued working	Unknown	Grade 2 SH

ACASI, Audio computer‐assisted self interviews; SH, social harm; IPV, intimate partner violence; ART, antiretroviral therapy; HIVST, HIV self‐testing; STAR‐GP: Self‐Test Africa Research – general population protocol; PASTAL, Partner Assisted HIVST and Linkage; CRS, community reporting systems; PSI, Population Services International; CRS, community reporting system; FSWs, female sex workers; FGD, focus group discussion; PRISM, Partnerships in Self‐Testing in Malawi.

The second type of coercion where individuals showing signs of ill‐health were persuaded to test to facilitate ART initiation was described as “compassionate coercion” (Table 4, Q4). Participants described these methods as often indirect. For instance, one man directed his household, including himself, to self‐test, but subsequently stated having done so out of concern for his orphaned nephew's health. Again, these instances tended to be viewed by individuals and the community as benign, and so unlikely to be spontaneously reported.

### 3.2 Verbal intimate partner violence

Arguments and verbal IPV, although infrequent, was more common following a reactive HIVST result, especially among couples and FSWs who disclosed their result. In these cases, ridicule, stigma, and blame tended to be directed towards the HIV‐positive partner in discordant couples, or the partner with suspected infidelity – usually the man – in an HIV‐positive concordant couples (Table 4, Q6 and Q7). These events were rarely reported, with participants tending to place blame on their partner, not the HIVST kit *per se*.

For FSWs, social stigma towards both users and peer distributors was reported. Some peer distributors reported they were insulted when distributing HIVST kits, particularly by FSWs who did not want to test (Table 4, Q8). Colleagues and neighbours also labelled some peer distributors as HIV positive, with others questioning their credentials and abilities to deliver HIVST (Table 4, Q9 and Q10).

### 3.3 Physical violence

ST‐Impacts followed up 150 women who reported physical violence to support organizations and identified 16 linked to HIVST. Of eleven women interviewed in‐depth, eight reported pre‐existing violence within their relationship, while three suffered violence for the first time after self‐testing (Table 4, Q11 and Q12). In seven of these cases, men refused to self‐test aggressively. Alcohol was a pre‐existent problem in the households of nine women. All women reporting violence were dependent on income from their male partner. While most women experiencing violence were aware that it was inappropriate, they often reported being disempowered or unable to prevent it due to inequalities in access to resources and normalized inequalities of power (Table 4: Q13). The most serious case resulted in hospitalization: here, the woman self‐tested negative prompting the man, who had been extremely violent in the past, to self‐test himself and become enraged when his result was positive. A second case of IPV, reported following self‐testing is detailed in Table 4 (Q13). No woman identified through critical incident interviews had been identified by the HIVST community representative system.

For FSWs, 92 of 268 women reported violent incidents but only two were confirmed to be HIVST‐related. In both cases, the incident was with established sexual partners and was similar to those reported in GP (Table 4, Q14 and Q15). There was also one instance of maltreatment of a peer distributor by a FSW who had a reactive self‐test result (Table 4, Q16).

### 3.4 Separation and break‐up

Four reconciled and four permanent marriage break‐ups resulted from HIVST (Tables 2 and 3), including seven serodiscordant couples. Marriage break‐ups tended to remain unreconciled in the early studies but were mostly reconciled in STAR‐GP. HIVST distributor training and sensitization of community‐led harms reporting system stakeholders (Figure 1.) included greater focus on serodiscordancy under STAR‐GP, where community‐led reporting systems identified the same number of marriage break‐ups (three couples) for standard‐of‐care villages as for HIVST villages.

Serodiscordant partnerships were more likely to report SHs when the woman was HIV positive (Table 4, Q17, Q18, Q19). A variety of misconceptions led serodiscordant couples to view their relationship as one that could not last: first, the concept was perplexing, with couples failing to understand how HIV could fail to be transmitted during condomless sex. Second couples were not aware that treatment‐as‐prevention could enable them to resume condomless sex once the positive partner was established on (and remained adherent to) ART (Table 4, Q20). Without this knowledge, couples assumed HIV must have been introduced recently (implying infidelity), and that condoms would be required indefinitely, precluding a healthy sex‐life or children (Table 4, Q21, for an HIV‐positive concordant woman). Importantly, correcting these misconceptions led to some couples reconciling.

Events unrelated to serodiscordancy were rare but included reactions to the mere introduction of an HIVST kit into the house without the man's permission, and occasional break‐up of concordant HIV‐positive relationships (Table 4, Q18).

### 3.5 Severe depression

One report of depression with suicidal ideation was documented within a recently formed serodiscordant relationship (Table 4, Q22). At 12 months, the HIV‐positive man still experienced suicidal ideation; however, this was related to specific financial worries. Three other cases of mild depression following disclosure and discrimination were reported in STAR‐GP.

### 3.6 HIVST age <16 years outside the study area

All studies restricted HIVST to those aged 16 or older; however, tests did find their way into non‐study areas. One case of self‐testing under the age of consent was identified by implementers in a non‐study area. In this case, a 12‐year‐old perinatally infected adolescent previously unaware of her status self‐tested with friends and experienced multiple serious SHs including psychological distress, stigmatization and economic upheaval (Table 5), illustrating the importance of training HIVST distributers to prevent HIVST kits to those aged under 16.

## 4 Discussion

In the past six years, over 175,000 HIVST kits have been distributed in urban and rural Malawi, with services implemented in settings characterized by high HIV prevalence, economic vulnerability and high frequencies of IPV. We adapted the grading system used for therapeutic clinical trials and post‐marketing pharmaceutical surveillance to classify both frequency and severity of SHs relating to HIVST. Despite the high levels of background SH, only 19 (0.011%) individuals involved in self‐testing or offering kits reported a serious SH related to HIVST, with multiple events affecting some individuals (25 serious SH events). Rates tended to be higher when kit recipients were followed up with interview for serious SHs, consistent with likely under‐reporting in less “active” surveillance systems 35, 36, 37, 38, for example 4 of 300 (1.3%) self‐tester from the general community, although no serious SHs were reported by 2349 pregnant women interviewed one month taking two HIVST kits home (Table 2). Our ability to comment on mild and moderate harms (defined as no/some effect on social and work life, respectively) was limited by the nature of data captured (Table 2), but the overall frequency of any reported SH from HIVST was within the range expected for standard HIV testing 36, 37, 38. For instance, 0.5% of 794 self‐testers included in post‐intervention household survey reported any unwanted consequences in rural Malawi. Most serious events related to the broader issues and challenges of being diagnosed and living with HIV in Africa particularly for those in serodiscordant relationships, rather than testing modality.

As previously reported, many couples considered HIVST to be a helpful tool to start dialogues and discussions on sensitive topics, including HIV testing, and a way to build trust between partners 5, 25. In general, women found the ability to bring kits home increased their autonomy and left them feeling empowered by testing themselves and offering HIVST to their male partners. Empowerment for women did leave some men feeling “coerced” to self‐test 6; however, most described it as well‐intentioned and socially acceptable within their established partnership 25, as also reported for men who have sex with men from China 19, 39. Nevertheless, several cases of coercion escalated into other forms of harm. It is important to reiterate that coercion and mandatory testing are never advised, including with HIVST 40. Programmes need to develop strong and clear messages to self‐testers and training for distributor to avoid overpressurising partners, especially when implementing index/partner‐delivered or network‐based distribution models, which encourage individuals to offer HIVST kits to sexual or social contacts with the endorsement of national health systems.

Because of high background rates of IPV among FSWs and previous reports of SH following HIVST 41, 42, 43, additional strategies to mitigate the risk of coercion and IPV among FSWs are needed. Approaches could include empowerment workshops and training for police and venue owners, which have been used more broadly in FSW programmes 44. Messages that explain FSWs rights to choose when and how to self‐test and disclosure, should be promoted. We found that high background IPV rates in FSWs made it difficult to directly relate events to HIVST, raising the need for additional methodologies or monitoring tools to better capture this information.

Couples with serodiscordant HIV results are an important target for HIV prevention in Africa, where serodiscordancy is common (e.g. 7% of Malawian couples jointly tested as part of the most recent Demographic and Health Survey 45 and transmission within serodiscordant couples accounts for a substantial fraction of all new HIV infections at the national level, and is readily preventable using ART‐based strategies. However, coping with newly identified serodiscordancy is challenging with any mode of HIV testing 36, 37, 38, 46. For instance, 24% of 469 serodiscordant Kenyan couples separating during two years of follow‐up in an HIV prevention trial 37, while in a multicountry East and Southern African trial 36, IPV was reported by 18% of HIV‐positive women and 7% of HIV‐positive men, respectively, in serodiscordant relationships.

Most serious and lasting SH identified across all HIVST studies reported here were also linked to newly identified serodiscordancy, making this a feature common to all HIV testing strategies 10, 11, 22. What is unique to HIVST, however, is the ease with which couples can self‐test together or soon after one another and share results. For this reason, HIVST appears to facilitate greater mutual knowledge of status between couples than other approaches 34, 47. There was anecdotal evidence in the PASTAL trial where 20 out of 46 male partners who self‐tested HIV positive were confirmed to be in an HIV‐discordant relationship. This presents an important opportunity for HIV prevention 48, but also responsibility to ensure that serodiscordancy is understood, with appropriate follow‐up advice and management. We identified significant gaps in awareness and understanding of discordancy, both among couples and for health workers, as also reported from other African countries 49, 50, 51. Providing clear messages and the need for further testing following a reactive self‐test result is a key to ensure partners are properly supported. Updating and disseminating national guidance to appropriately address the needs for serodiscordant couples should be prioritized 11. In this context, each of the three newly identified discordant couples who separated following HIVST and were provided with information and support under the community‐led system of the STAR general population study recovered their relationship, whereas none of the three discordant couples who separated following standard HIV testing and counselling (HTC) in the control villages did so (Tables 2 and 5).

Although not captured in our matrix, SH reported by distributors also need to be anticipated, as many programmes will be reaching out to groups that experience stigma, discrimination and criminalization. We found that distributors delivering HIVST kits to FSWs experienced interpersonal violence, and stigmatizing and discriminatory attitudes. Programmes need to consider the context where they are implementing and identify ways to address these types of issues, and consider training distributors on techniques for avoiding and de‐escalating conflict. Where feasible, community consultations should also be considered.

Monitoring SH is challenging, particularly for HIVST. Our findings suggest that community‐led approaches are feasible, but subject to under‐reporting. While intensive research methods identify more incidents, these approaches are not feasible for national programmes rolling‐out HIVST. It will be important to share programmatic experiences to optimize and integrate SHs reporting into existing monitoring systems and to focus on methods that can be scaled‐up. These approaches should also consider ways to identify and quantify social benefits, since this will help understand the broader social impact of HIVST at the individual and community levels.

Strengths of this study include the use of community‐based reporting systems combined with in‐depth qualitative and mixed methods to identify and understand SHs in the context of HIVST. Limitations include that our proposed harms grading system was developed iteratively, built on established pharmacovigilance methods to grade severity according to patient‐centred criteria, and broadened from an initial focus on IPV and partnership dissolution. As such, data from earlier studies could not be completely mapped. Secondly, we do not have estimates of the numbers of newly identified serodiscordant couples who managed their relationship without separation, except for the smaller urban studies. To estimate the number of HIVST episodes, we used the total number of HIVST kits distributed as a proxy. Although we cannot define exact usage, participants receiving kits through community‐based distributors were asked to return their used kits with a self‐administered questionnaire. Use was confirmed by inspection of used kits for 75.7% of 27,789 distributed kits in HitTB (Study 1, Table 2) and 53.2% of 163,300 kits distributed under STAR‐GP in Malawi 52 including 137,915 kits for which we report SHs (Studies 9 and 11, Table 2) 4, 6, 52. Thus, while non‐use of distributed kits will be contributing to underestimation of SHs, this has relatively little impact on our SH frequency‐estimates reported here. For example, if true kit use was as low as 53%, then serious SH frequency would increase to 19/95,228 or 0.02%. Also, as return‐and‐reread of kits is not practical during routine implementation internationally recommendations are to report HIVST metrics based on kits distributed 53. Finally, the studies presented here are from a single country, where background rates of IPV are high and the HIVST distribution models were primarily community‐based and partner‐delivered HIVST.

## 5 Conclusions

Six years of large‐scale HIVST implementation and in‐depth investigation in Malawi identified no reported suicides and report of serious SHs to be rare. SH incidents reported mainly related to identification of serodiscordant HIV results within established relationships. Resolution tended to draw on existing structures, including community reporting. As access to HIVST increases, programmes need simple messages about both coercion and discordancy, urging restraint even when coercion is well‐intentioned or “compassionate,” and stressing the preventative benefits of treatment for serodiscordant couples.

Specific consideration must be given to HIVST programmes for FSW to make sure that distribution methods are safe and appropriate, and that clients or employers are not involved. It is also important that HIVST is available only to those who are of appropriate and legal age of consent to test. Continued efforts are needed to mitigate potential risks, optimize HIVST distribution and to monitor SHs and benefits following HIVST.

## Competing interests

We have no competing interests to declare.

## Authors’ contributions

MK, CJ, ND and ELC drafted the manuscript with inputs from all authors. MT led the qualitative research network in STAR and contributed to the qualitative analysis. MK designed the PRISM study while ND and WL developed the ST‐Impacts study. AC designed the PASTAL trial supported by MK and DS. ELC and PI led the research for STAR project in Malawi and were supported by WS and MK. KH and RC led the STAR implementation. All authors contributed to the final manuscript.
